# Novel Vanadium-Loaded Ordered Collagen Scaffold Promotes Osteochondral Differentiation of Bone Marrow Progenitor Cells

**DOI:** 10.1155/2016/1486350

**Published:** 2016-05-11

**Authors:** Ana M. Cortizo, Graciela Ruderman, Flavia N. Mazzini, M. Silvina Molinuevo, Ines G. Mogilner

**Affiliations:** ^1^LIOMM, Dto. Ciencias Biológicas, Facultad de Ciencias Exactas, Universidad Nacional de La Plata, 1900 La Plata, Argentina; ^2^IFLYSIB, CONICET, Facultad de Ciencias Exactas, Universidad Nacional de La Plata, 1900 La Plata, Argentina

## Abstract

Bone and cartilage regeneration can be improved by designing a functionalized biomaterial that includes bioactive drugs in a biocompatible and biodegradable scaffold. Based on our previous studies, we designed a vanadium-loaded collagen scaffold for osteochondral tissue engineering. Collagen-vanadium loaded scaffolds were characterized by SEM, FTIR, and permeability studies. Rat bone marrow progenitor cells were plated on collagen or vanadium-loaded membranes to evaluate differences in cell attachment, growth and osteogenic or chondrocytic differentiation. The potential cytotoxicity of the scaffolds was assessed by the MTT assay and by evaluation of morphological changes in cultured RAW 264.7 macrophages. Our results show that loading of VOAsc did not alter the grooved ordered structure of the collagen membrane although it increased membrane permeability, suggesting a more open structure. The VOAsc was released to the media, suggesting diffusion-controlled drug release. Vanadium-loaded membranes proved to be a better substratum than *C*0 for all evaluated aspects of BMPC biocompatibility (adhesion, growth, and osteoblastic and chondrocytic differentiation). In addition, there was no detectable effect of collagen or vanadium-loaded scaffolds on macrophage viability or cytotoxicity. Based on these findings, we have developed a new ordered collagen scaffold loaded with VOAsc that shows potential for osteochondral tissue engineering.

## 1. Introduction

Osteochondral damage is a frequent consequence of traumatic and degenerative alterations of joints and bones [1]. As articular cartilage is an avascular tissue in which differentiated cells are embedded in an organic matrix, it has a very limited potential for spontaneous healing [2, 3]. Different tissue engineering-based strategies have been used to improve the regenerative capacity of osteochondral lesions. In particular, a microfracture procedure combined with scaffold implantation with or without the addition of mesenchymal stem cells and/or growth factors has shown promising results [4–7]. Filling the osteochondral defect with an adequate matrix substitute (i.e., with functional characteristics similar to those of the original tissue) can decrease reparation time and thus patient morbidity [7–9]. The use of mesenchymal stem cells is of particular interest since in response to specific factors they present the ability to differentiate into osteoblasts or chondroblasts and since they can be obtained from the same patient [10]. Despite these encouraging aspects, an important number of patients submitted to these procedures show worsening of their lesions, underscoring the importance of perfecting the design of an adequate biomaterial.

The inclusion of different bioactive molecules for bone and/or cartilage regeneration has also been reported, leading to the design of scaffolds that can function as a controlled delivery system [11, 12]. This could improve the osteoinductive properties of the matrix and even reduce the cytotoxicity of a certain drug, thus reducing the time required for tissue repair. In addition, designing a functionalized or “intelligent” biomaterial could optimize the degradation rate and dosification of the bioactive molecule.

Although many materials have been developed for the repair of osteochondral defects, none has equalled the properties of collagen [13]. This natural polymer has been used extensively because of its inherent biocompatibility, biodegradability, osteoconductivity, and cost-effective availability [14]. Recently, we have prepared and characterized a microscale ordered collagen scaffold and shown its efficacy for bone tissue engineering [15]. Our results suggest that the orientation of collagen fibers positively regulates osteoblastic growth and development. When compared to a randomly oriented collagen scaffold, the ordered collagen matrix enhanced the in vitro osteogenic differentiation of MC3T3E1 preosteoblasts increasing markers of osteoblast differentiation and extracellular matrix mineralization [16].

In addition, we have previously reported the osteogenic properties of a vanadyl(IV) ascorbate (VOAsc) complex as well as its possible mechanisms of action, on two osteoblastic cell lines in culture [17]. VOAsc significantly stimulated osteoblastic proliferation and type I collagen production and increased the formation of mineralization nodules. We demonstrated that this complex inhibited several phosphatases, while simultaneously activating the ERK pathway and regulating intracellular calcium levels and the PI3-kinase pathway. Altogether our observations suggest that the vanadium(IV) ascorbate complex could be a useful pharmacological tool for bone tissue regeneration. On the other hand, we have evaluated a delivery system for vanadium using poly(*β*-propiolactone) films [18]. In this system, vanadium was liberated at a controlled rate causing a selective antiproliferative effect on osteosarcoma cells with lower cytotoxicity than the drug in solution.

Based on our previous data, in the present work we have designed an ordered collagen scaffold loaded with different amounts of VOAsc and evaluated its potential use for regeneration of bone and cartilage. We have determined several physicochemical and biocompatibility properties of the collagen scaffolds. In particular, we have investigated the effects of VOAsc loaded into the membranes on the growth of rat bone marrow progenitor cells (BMPC) and on their phenotypic differentiation into osteoblasts and chondroblasts. We have also evaluated the possible in vitro cytotoxicity of the scaffolds.

## 2. Materials and Methods

### 2.1. Membrane Preparation and Characterization

Membranes were prepared using acid-soluble collagen extracted from bovine Achilles tendon. Ordered films were obtained according to standardized procedures of our laboratory [15]. Vanadyl(IV) ascorbate (VOAsc) was synthesized as we have previously described and characterized by UV-vis and infrared spectroscopy [17, 19]. Stock solutions were prepared in distilled water at room temperature and used immediately for the different assays. Membranes of pure collagen were loaded with the VOAsc complex and given an ordered pattern by layering the collagen and vanadium solutions (50, 100, or 200 *μ*g/mL) alternatively on a mold, according to our previously described method [15]. This formulation was selected in order to obtain 10, 20, or 40 *μ*g/mL VOAsc per cm^2^ of membrane surface (*C*1, *C*2, and *C*3). An ordered collagen membrane without vanadium was also prepared as a control (basal condition, *C*0).

Surface characteristics of the membranes were investigated using scanning electron microscopy (SEM; Phillips 505, Netherlands), with an accelerating voltage of 25 kV. The images were analyzed by Soft Imaging System ADDAII. Fourier Transformed Infrared (FTIR) spectra were collected to analyze and compare the material characteristics of all scaffold samples following VOAsc addition. FTIR analysis was carried out using an IRAffinity-1 Spectrum FTIR (Shimadzu). Scaffolds were cut and fixed in a port-cell and spectra were collected between 2000 and 800 cm^−1^.

Permeability was evaluated by measuring the flux of NaCl across the membranes, with a custom-made glass cell as previously described [15]. In this device the membrane divides the cell in two compartments: one of them (*L*) was filled with the solution under study and the other (*R*) with distilled water. The complete cell was kept in a thermostatic bath at 37°C. The two half cells were stirred with a magnetic device to avoid the possible formation of an unstirred layer on the membrane surface. Fluid samples were taken from compartment *R* at regular intervals for conductivity determination of NaCl concentration with a Radiometer CMD3 conductivity meter. A calibration was previously performed. Permeability is given by

(1) $$\begin{array}{c} P=V\Delta \left(\;-\frac{\ln\left(1-2C\left(t\right)/C\left(0\right)\right)}{2a\Delta t}\;\right), \end{array}$$

where *V* is the cell volume, *a* the membrane area, *C*(0) the concentration of the solution at *t* = 0, and *C*(*t*) the concentration at time *t*. A plot of ln⁡(1 − 2*C*(*t*)/*C*(0)) against *t* was made using the determinations of *C*. After a least square fit, the slope was taken as the value of *P*.

### 2.2. Vanadium Release Kinetic Studies

The release profile of VOAsc from the scaffold was determined by incubating scaffold samples (1 cm^2^) loaded with different amounts (10, 20, and 40 *μ*g/mL per cm^2^) of VOAsc in 1.0 mL of sterile DMEM without phenol red (pH = 7.4) at 37°C for different periods of time. At appropriate times (every 15 min during the initial hour, then every 30 min until 5 h, and finally every hour for 24 h), the supernatant was removed and replaced by fresh media. The time-dependent release of the drug was followed by monitoring the amount of VOAsc present in the supernatant medium, using a T60 UV-visible spectrophotometer (PG Instrument). A linear calibration curve of VOAsc concentration versus absorbance at 580 nm was obtained using VOAsc standards in the range 0-1 mg/mL. The assay was performed in quadruplicate and results were expressed as the fractional release (*M*_*t*_/*M*_*∞*_) versus time of release (*t*).

### 2.3. Biocompatibility Studies

#### 2.3.1. Cell Cultures and Incubations

The biocompatibility of Col and Col-V (loaded with different amounts of VOAsc) membranes was evaluated using rat bone marrow progenitor cells (BMPC), investigating possible changes in their morphology, growth, osteoblastic induction, and chondrogenic differentiation when grown on the different scaffold. BMPC were chosen because they represent a better physiologic model of osteogenesis due to its ability of self-renewal and multilineage differentiation compared to cloned established cell lines [20]. Under appropriate conditions, these cells are able to differentiate into osteoblasts, chondroblasts, or adipocytes. Additionally, they could also be used for in vivo studies of bone tissue regeneration. BMPC were isolated from the femora of young male Sprague-Dawley rats and cultured as previously described [21]. Cells were maintained in a basal medium (DMEM-10% FBS) at 37°C until being plated on the different membranes, after which fresh medium was added every 2 days. Prior to their use for cell culture, 1-2 cm^2^ scaffold samples were sterilized by immersion in 70% ethanol and irradiation with UV light. After different incubation periods, the *C*0 and Col-V vanadium-loaded membranes were processed to evaluate cell adhesion, proliferation, or differentiation.

**Figure 1:**
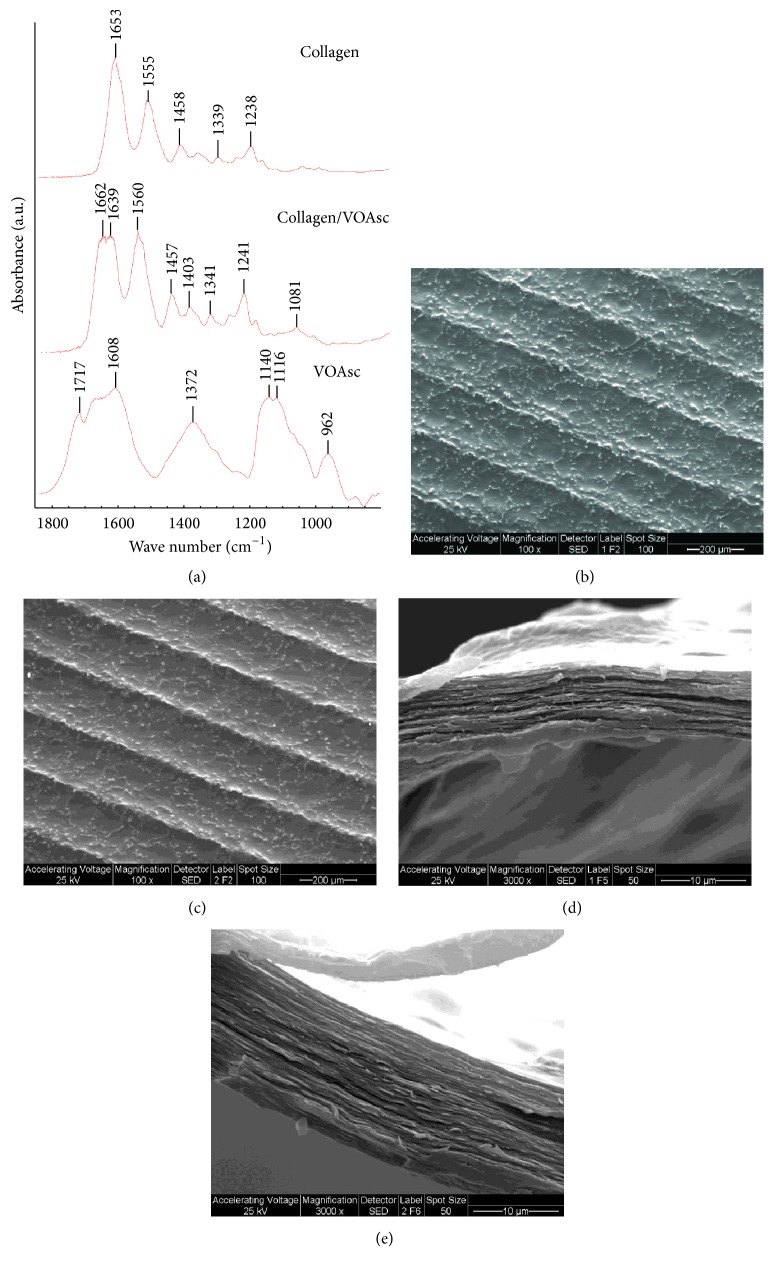
Characterization of Col and Col-V scaffolds. FTIR spectra of Col, Col-V (20 *μ*g/mL per cm^2^ VOAsc) membrane, and the VOAsc complex (a). SEM images of Col and Col-V surfaces ((b) and (c), resp.) showed an ordered matrix with a grooved structure. Transversal section of Col and Col-V films ((d) and (e), resp.).

**Figure 2 fig2:**
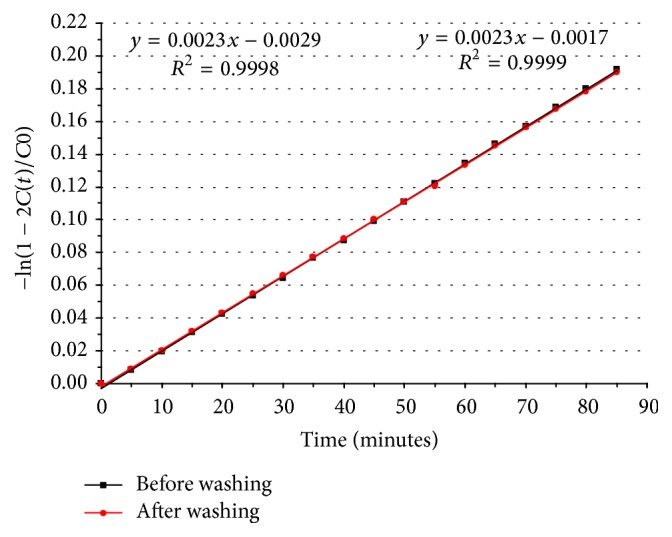
NaCl flux experiments across Col-V (20 *μ*g/mL per cm^2^ VOAsc) membrane at a temperature of 37°C before (black) and after (red) a 24 h wash of the membrane in distilled water.

**Figure 3 fig3:**
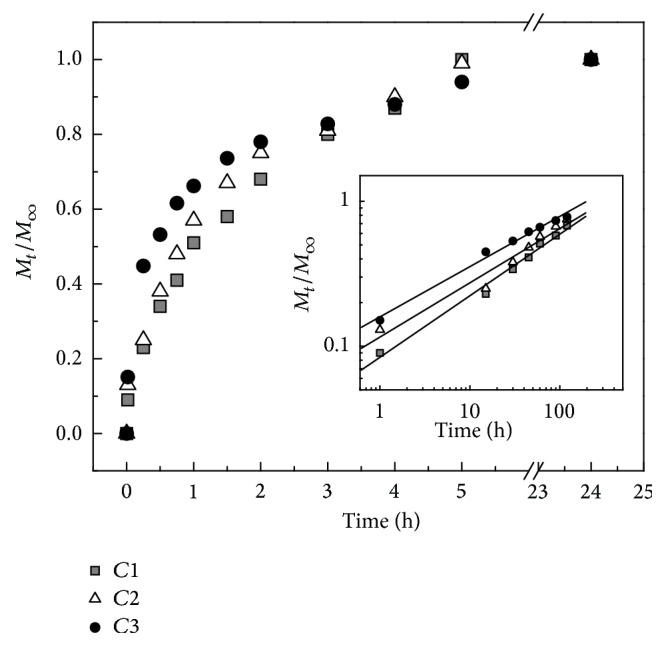
Fractional VOAsc drug release from the Col-V membranes in vitro. *C*1: 10 *μ*g/mL per cm^2^ VOAsc; *C*2: 20 *μ*g/mL per cm^2^ VOAsc; and *C*3: 40 *μ*g/mL per cm^2^ VOAsc. Release was measured up to 24 h. Insert: plot of log⁡*M*_*t*_/*M*_*∞*_ versus log⁡*t* at initial times. Data are presented as mean (*n* = 3).

**Figure 4 fig4:**
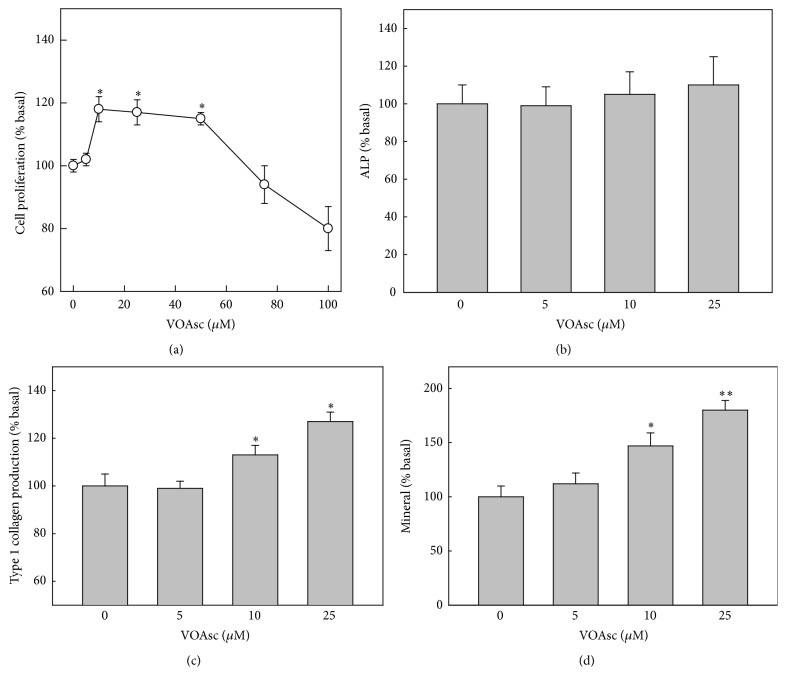
Effect of VOAsc on cell proliferation, differentiation, and mineralization. BMPC were incubated with different doses of VOAsc. Cell proliferation was determined after 24 h of culture (a) and osteoblastic differentiation was assessed by ALP (b), type 1 collagen production (c) and matrix mineralization by calcium nodules deposition (d) after 2 weeks of culture in an osteogenic media in the presence of different concetrations of VOAsc. Data represent the mean ± SEM of three independent experiments and are expressed as % basal. ^*∗*^ *p* < 0.05, ^*∗∗*^ *p* < 0.01.

**Figure 5 fig5:**
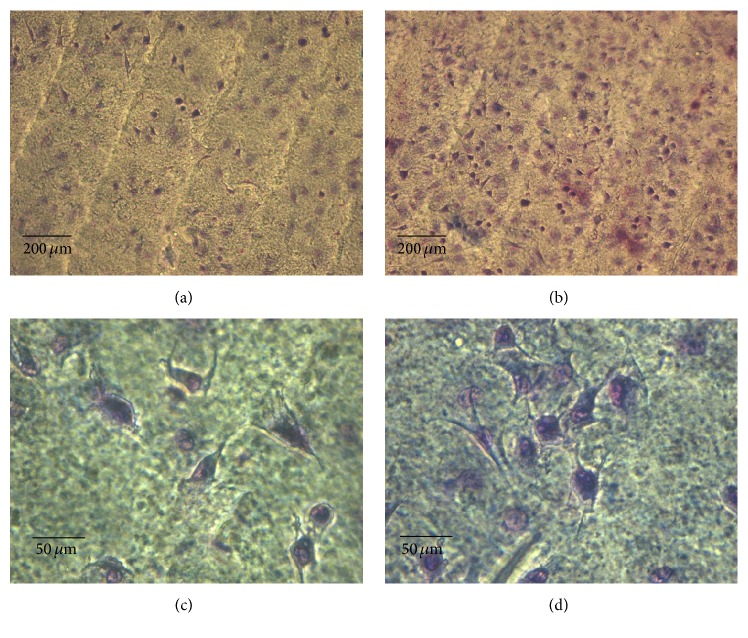
BMPC attachment (2 h at 37°C) to *C*0 ((a) and (c), Obj. ×10.) and *C*2 (20 *μ*g/mL per cm^2^ VOAsc) membranes ((b) and (d), Obj. 40x). Giemsa staining. Morphological images of BMPC are also representatives of the results for *C*1 and *C*3.

**Figure 6 fig6:**
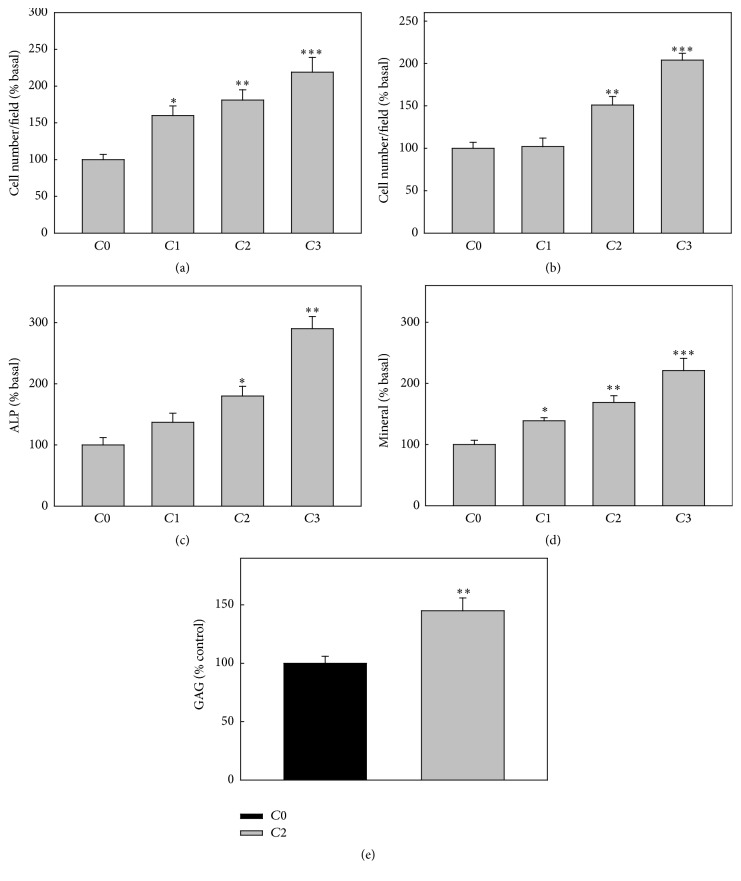
Biocompatibility of BMPC on *C*0 and VOAsc-loaded scaffolds: *C*1: 10 *μ*g/mL per cm^2^ VOAsc; *C*2: 20 *μ*g/mL per cm^2^ VOAsc; and *C*3: 40 *μ*g/mL per cm^2^ VOAsc. Cells were plated on different collagen membranes and adhesion (1 h (a)) and proliferation (24 h (b)) were evaluated by Giemsa staining. Cells in 10 representative fields per sample were counted and averaged. BMPC growing on different membranes were induced to be differentiated in osteogenic media: ALP (15 days (c)) or mineralization (21 days (d)) or chondrogenic media, GAG production (21 days (e)). Results are expressed as % of basal (cells differentiated in the *C*0 film) and they are expressed as mean ± SEM. ^*∗*^ *p* < 0.05; ^*∗∗*^ *p* < 0.01; ^*∗∗∗*^ *p* < 0.001.

**Figure 7:**
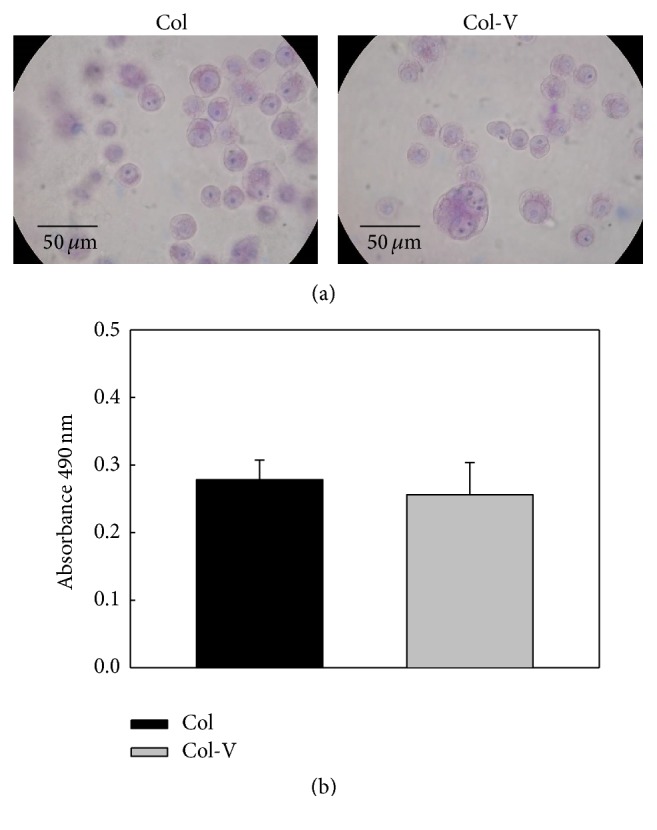
Cytotoxicity studies with RAW 264.7 macrophages. (a) Cells were cultured for 24 h on Col or Col-V (20 *μ*g/mL per cm^2^ VOAsc) scaffolds, stained with Giemsa and photographed. (b) An MTT assay was performed to evaluate cell survival of BMPC growing on Col or Col-V scaffolds. Results represent the mean ± SEM.

In order to compare the direct effect of VOAsc addition in the cell culture media with the effects of the compound released from the membranes, some experiments were performed with the BMPC plated on standard tissue culture plates. In these cases, cells were incubated in a basal medium with different doses of VOAsc complex in solution as indicated in Figures 1 [Fig fig2] [Fig fig3] [Fig fig4] [Fig fig5] [Fig fig6]–7. After different incubation periods, cell monolayers were processed to evaluate proliferation and osteogenic differentiation as described below.

#### 2.3.2. Evaluation of Cell Growth

The cells were washed with phosphate-buffered saline (PBS, pH 7.4), after which adherent cells were fixed with methanol, stained with Giemsa, observed using a TS100 Eclipse Nikon microscope, and photographed with a CCD camera with a 0.7x DXM Nikon lens [16]. Cell adhesion (1 h after seeding) and proliferation (24 h) were evaluated by counting the number of cells/field in 10 representative fields per experimental condition.

#### 2.3.3. BMPC Differentiation

For BMPC osteoblastic differentiation, cells were incubated for different periods of time in osteogenic media (10% FBS-DMEM supplemented with 5 mM *β*-glycerol-phosphate and 25 *μ*g/mL ascorbic acid). Osteogenic differentiation was evaluated by alkaline phosphatase specific activity (ALP) and extracellular calcium deposition (mineralization nodules). For ALP determination, cells submitted to a 15-day osteogenic differentiation were washed with PBS and solubilized in 0.5 mL 0.1% Triton X-100. Aliquots of this total cell extract were used for protein determination [22] and for measurement of ALP by spectrophotometric determination of the initial rates (10 min) of hydrolysis of p-nitrophenyl-phosphate to p-nitrophenol at 37°C. Mineralization nodules were measured after 21 days of osteogenic differentiation using Alizarin S red staining. Stained calcium deposits were extracted with 1 mL of 0.1 N sodium hydroxide and the optical density recorded at 548 nm. Additionally, type 1 collagen production was evaluated in the cell monolayer plated into the standard tissue culture plate to assess the effect of VOAsc in solution, by Sirius red staining, as we previously described [17].

Chondrogenic differentiation was assessed after a 21-day culture in a chondrogenic medium [23]. Briefly, 10^7^ BMPC/mL were resuspended in 40 *μ*L of serum-free DMEM, and four individual drops (10 *μ*L per drop) were carefully placed on Col or Col-V films included in each well interior of 24-well plates. Cells were allowed to adhere at 37°C for 2 h. Basal medium was then added for an additional 24 hours, after which it was replaced by a chondrogenic medium (serum-free DMEM supplemented with 10 ng/mL of TGF-b3 (PeproTech, USA), 10^−8 ^M dexamethasone, and 1x insulin transferring selenium (ITS) supplement (Invitrogen)), in which cells were cultured for 21 days changing the medium every three days. Production of chondroitin sulphate glycosaminoglycan (GAG), a marker of chondrocytic differentiation, was evaluated by Alcian blue staining (pH 3) at the end of the culture period. Briefly, cells were fixed overnight and stained with 0.5% Alcian blue in 0.1 N HCl, rinsed twice with 0.1 N HCl, and once with distilled water. Finally, the dye was extracted with 4 M guanidinium HCl and absorbance was measured at 600 nm.

### 2.4. Evaluation of Scaffold Cytotoxicity

RAW 264.7 monocyte-macrophage cells were maintained in DMEM containing 10% FBS, 100 U/mL penicillin, and 100 *μ*g/mL streptomycin at 37°C in a 5% CO_2_ atmosphere. This cell line has previously been used in our laboratory to assess cytotoxicity since it represents an adequate and sensitive in vitro model for inflammation [16, 24]. After subculturing with 10% EDTA, cells were seeded on either the Col or Col-V membranes and incubated for 24 h after which the MTT bioassay was performed. Briefly, 5 mg/mL of MTT solution was added and incubated for 3 h [24] until formation of purple formazan crystals, which were dissolved in dimethyl sulfoxide (DMSO) and absorbance measured at 490 nm. In other experiments, morphology of RAW 264.7 cells cultured on either scaffold was analyzed after Giemsa staining, using a TS100 Eclipse Nikon microscope, and photographed with a CCD camera with a 0.7x DXM Nikon lens.

### 2.5. Statistical Analysis

Results are expressed as the mean ± SEM and were obtained from at least three separate experiments. Differences between the groups were assessed by one-way ANOVA using the Tukey post hoc test. For nonnormal distributed data, the nonparametrical Kruskal-Wallis test with Dunn post hoc test was performed, using GraphPad InStat, version 3.00 (GraphPad Software, San Diego, CA, USA). *p* < 0.05 was considered significant for all statistical analyses.

## 3. Results

### 3.1. Membrane Characterization

Inclusion of vanadium in the scaffold was demonstrated by FTIR spectra. The presence of VOAsc in the collagen scaffold was evidenced by the characteristics peaks for ascorbic acid at 1755 cm^−1^ (C=O stretching) and 1670 cm^−1^ (C=C vibration) as has been previously described [19]. In the Col-V membrane spectra, both a characteristic strong band of *ν*(V=O) appearing at 962 cm^−1^ and a peak at 1372 cm^−1^ corresponding to the (C3-O-)V complex were observed. In addition, characteristic peaks for collagen were evidenced at 1653 cm^−1^ (Amide I), 1550–60 cm^−1^ (Amide II), and 1240 cm^−1^ (Amide III) (Figure 1(a)).

SEM analysis demonstrated a scaffold surface with parallel alignment of collagen fibers and a grooved structure (Figure 1(b)), which was not influenced by the presence of vanadium complex (Figure 1(c)). Superposition of the collagen layers was homogeneous on both Col (Figure 1(d)) and Col-V (Figure 1(e)) scaffolds, revealing no influence of vanadium complex on the morphology of the layers, as observed by SEM. However, since SEM is not the most sensitive method to evaluate alterations in pore structure of the scaffold we also investigated the permeability properties of the membranes.

Permeability is an important physicochemical characteristic since VOAsc must be delivered from the scaffold in order to act as an osteogenic compound. We measured the flux of NaCl across the membrane as a measure of its permeability at 37°C. We found that the permeability value of Col-V membrane was 2.16 × 10^−4 ^cm/sec, which represents an increase of about 40% compared to our previously described values for Col scaffold (1.47 × 10^−4 ^cm/sec [15]) (Figure 2). We also evaluated the possible effect of VOAsc delivery on scaffold permeability. Thus, permeability of Col-VOAsc scaffolds was determined initially as described above and again using the same membrane after 24 h of washing in distilled water (Figure 2 red plot). We found an overlap for both curves, indicating no differences in permeability coefficient.

### 3.2. VOAsc Release Kinetics

In order to analyze the kinetics of vanadium release, we used Fick's second law for one-dimensional transport in thin polymeric films with a moderate swelling rate [25]:

(2) $$\begin{array}{c} \frac{M_{t}}{M_{\infty }}=kt^{n}, \end{array}$$

where *M*_*t*_ is the cumulative absolute amount of drug released at time *t*; *M*_*∞*_ is the absolute cumulative amount of drug profile released at infinite time; *k* is a constant incorporating structural and geometric characteristics of the device; and *n* is the release exponent, indicative of the mechanism of drug release.  Figure 3 shows the fractional vanadium release from *C*1 to *C*3 films (containing different concentrations of the drug) as a function of time. As it can be observed, the VOAsc complex was released to the media in a controlled manner, with a fast and linear kinetic during the first 3 h. At initial rate a slight difference could be observed in the VOAsc release for the different vanadium-loaded membranes. Nevertheless, at the end of the incubation time, a saturation curve was observed for each membrane with the release of vanadium complex sustained for 24 h. The kinetics of such process could be analyzed through the Fick model in order to determine the *n* exponent. The insert in Figure 3, a plot of log⁡*M*_*t*_/*M*_*∞*_ versus log⁡*t*, shows the linear regression plots of the fractional vanadium release at short times. The diffusion coefficient values (*n*) were 0.425 ± 0.02, 0.374 ± 0.03, and 0.347 ± 0.02 for *C*1, *C*2, and *C*3, respectively, although not statistically different. These results suggested that the mechanism of vanadium release occurs by a Fickian diffusion process.

### 3.3. Effects of VOAsc on BMPC Proliferation and Differentiation

In a first series of experiments, we used BMPC seeded on standard tissue culture plates, in order to investigate the effects of the VOAsc complex added directly into the culture media. Treatment of cells with 2.5–100 *μ*M VOAsc for 24 h led to a biphasic effect on cell proliferation.  Figure 4(a) shows that 10–50 *μ*M VOAsc significantly stimulated BMPC proliferation, while higher doses showed a tendency to inhibit cell growth. The effect of vanadium complex on the BMPC osteogenic potential was assessed after 2 weeks of culture in osteogenic media, by determination of ALP, type 1 collagen production, and nodules of mineral. As can be seen in Figure 4(b), VOAsc did not affect ALP in BMPC. Under similar conditions, VOAsc dose-dependently increases type 1 collagen production and calcium deposition in mineralized nodules. These results indicate that, at low doses, VOAsc is a weak mitogen and that its long term exposure to BMPC results in osteogenic effects.

### 3.4. Biocompatibility of VOAsc-Collagen Scaffolds on BMPC

Biocompatibility of the scaffolds was investigated using BMPC. Cells were seeded on *C*0 and *C*1 to *C*3 scaffolds and allowed to adhere (2 h) or growth (24 h). Cells attached to both *C*0 and *C*2 membranes showed a random distribution with homogeneous attachment between crests and valleys (Figures 5(a) and 5(b), resp.). At higher magnification, cells were either rounded or polyhedral with one or more cytoplasmic extensions (Figures 5(c) and 5(d)). Similar results were obtained for the other VOAsc-loaded membranes, *C*1 and *C*3 (data not shown).

Then, we investigated the effect of loading different doses of VOAsc on the collagen membranes in BMPC growth and differentiation. As it can be seen in Figures 6(a) and 6(b) there was a doses-dependent increase on both cell adhesion and proliferation of BMPC compared to the ordered collagen membrane (*C*0). Cell differentiation was also improved in the vanadium-loaded matrices. In those scaffolds, there was a dose-dependent increase on ALP activity (Figure 6(c)) and mineral deposits (Figure 6(d)) compared to collagen ordered matrix (*C*0). In addition, cells differentiated to chondrocytes produced more GAG on *C*2 (20 *μ*g/mL VOAsc per cm^2^) scaffolds than on the *C*0 membranes (145 ± 11% of Col, Figure 6(e)).

Thus, our observations suggest that the addition of a growth factor such as the vanadium(IV) ascorbate complex can improve the ability of a collagen matrix to support adhesion, growth, and differentiation of BMPC to an osteoblastic and chondrocytic phenotype.

### 3.5. Cytotoxicity Studies

Although collagen has been previously used for scaffold preparations and it demonstrated being biocompatible, we wondered if our constructs might generate any cytotoxic effect on cultures of RAW 264.7 macrophages.  Figure 7(a) shows these cells' growth well on both the *C*0 and *C*2 scaffolds, maintaining their round monocytic morphology without any signs of activation (i.e., absence of cytoplasm expansions such as spreading or formation of cell protrusions). MTT evaluation demonstrated the same number of surviving cells growing on *C*0 and *C*2 membrane (Figure 7(b)).

## 4. Discussion

Many strategies for bone tissue repair have focused on the development of biomimetic scaffolds formulating innovative tissue substitutes by the synergistic combination of matrices with cell therapy. An interesting strategy is the combination of synthetic and natural polymers in order to achieve suitable mechanical properties for bone tissue. Additionally, improved osteoconductivity can be accomplished by the incorporation into the scaffold of growth stimulating substances and/or stem cells. Collagen is the principal component of bone extracellular matrix and constitutes an interesting material for bone tissue engineering since it provides the innate biological information required for cell adhesion, proliferation, and orientation and promotes a chemostatic response [26]. On the other hand, collagen can be easily oriented to obtain scaffolds with a micro- or nanometric structure, which have been demonstrated to act as templates for osteoblastic development [15, 16]. In this study, we have extended our previous research to design a collagen-based scaffold that incorporates a vanadium complex (VOAsc) with in vitro osteogenic properties. Inclusion of VOAsc into the collagen matrix did not influence the macrostructure of the scaffold as observed by SEM, although it did increase the permeability of the biomaterial. Interestingly, there was no alteration in the permeability of vanadium-loaded scaffolds before and after 24 h of washing, even when delivery experiments demonstrated that VOAsc was positively released to the media in the same period of time. Thus, we hypothesize that the vanadium complex interacts reversibly with the collagenous matrix creating a more permeable structure but does not lodge in the pores of the matrix. Permeability is a physical parameter that describes complicated properties of a material regulating various aspects of molecule transport inside and outside the scaffold. This property is also an important regulator of cell differentiation and scaffold degradation [27–29]. Higher permeability values appear to be required for osteogenic versus chondrogenic marker expression, an effect correlating with the lower oxygen tension needed for maintenance of chondrocytic phenotype [27]. In our culture conditions, BMPC were able to grow and differentiate into osteoblasts or chondrocytes on ordered collagen scaffolds, expressing adequate levels of osteogenic or chondrocytic markers when differentiated in lineage-specific conditions. However, since both chondrogenic and osteogenic induction were significantly increased in the more permeable vanadium-loaded scaffold, permeability does not seem to be a primary regulator of cell fate in our system.

To date, several studies have evaluated bone and cartilage regeneration using collagen scaffolds [5, 29, 30]. In these studies, collagen constructs have been found to effectively repair articular cartilage or bone defects. However some unsolved problems remain, such as the need for periosteal scaffold implantation to prevent detachment, articular substitution of hyaline cartilage by fibrocartilage, persisting pain, and inadequate mechanical properties [5, 29–31]. In order to improve the osteoinductivity of our ordered collagen matrix we loaded it with the osteogenic complex VOAsc. We have previously demonstrated that VOAsc in solution stimulates osteoblastic growth and development, increasing collagen secretion and extracellular mineralization in MC3T3E1 cells. These effects correlated with an activation of the extracellular regulated kinase (ERK) pathway [17]. We also shown that VOAsc induces a fast ERK phosphorylation (minutes to hours) and redistribution, suggesting that this complex could regulate osteoblastic growth by MAPK pathways. In our present study, we confirm previous observation of VOAsc by using rat BMPC. BMPC are characterized by self-renewal capacity and multilineage differentiation under specific culture conditions. Additionally it represents a better model of bone cells, with the advantage that can be used in both in vitro and in vivo studies for bone tissue regeneration. The direct addition of VOAsc to the culture media stimulated proliferation of BMPC growing on standard tissue culture plates in a biphasic manner. Although low doses (10–50 *μ*M) significantly enhance cells growth, higher ones could be inhibitory. In long term experiments, low doses of VOAsc (5–25 *μ*M) significantly enhanced osteogenic potential of BMPC, as indicated by osteoblastic markers type 1 collagen production and matrix mineralization. More importantly, these results suggest nontoxic effect of the vanadium complex on these cells.

In our present system (BMPC grown on collagen scaffolds) it is to be expected that VOAsc must first be released from the scaffold to exert cellular actions. Thus the release kinetics was evaluated, showing a fast VOAsc release within the first three hours of matrix immersion into the culture media, after which the release reached a plateau. This effect was shown not to be dependent on the concentration of VOAsc loaded in the Col scaffold (Figure 3). Besides, the evaluation of diffusion coefficient for the three membranes studied was very similar suggesting that the rate of release kinetic is mainly dependent on the diffusion process. Our results could also be indicating that while an initial greater release of VOAsc might benefit BMPC attachment and growth, lower but sustained release of the vanadium complex thereafter could be acting as an osteogenic or chondrogenic agent. In this context, we found a stimulation of ALP activity on cell growing on Col-V scaffolds in a dose-dependent manner. The direct addition of VOAsc in the media did not modify this marker. Alternatively, the initial interaction of VOAsc present in the membrane with the cells could initiate a signaling to induce specific transcriptor factors associated with the fate of BMPC. Similar suggestions have been proposed by Laurencin's group by the short-term treatment of small osteogenic molecules [32]. Local delivery has emerged as an alternative to systemic delivery as it can avoid adverse drug effects. In this sense other groups have developed controlled-release systems directed to bone tissue, finding both a decrease in drug toxicity and an increase in bone and cartilage matrix deposition [33–35]. Indeed, inclusion of an osteogenic drug in the scaffold would be expected to diminish repair time [12].

Another important matter of concern is the possibility that the implantable scaffolds may cause toxicity, inflammation, or immunogenicity. For this reason we performed experiments to evaluate the response of a macrophage cell line in culture, RAW 264.7 cells, which is an accepted model to study inflammatory response and cytotoxicity of drugs or scaffolds. We have previously demonstrated that some vanadium complexes directly added to culture media can cause toxic effects such as an increase in reactive oxygen and nitrogen species [36, 37]. In addition, oral treatments with some vanadium derivatives have shown gastrointestinal toxicity [38]. In this work, our results demonstrated that there were no morphological changes in cells growing on either collagen or vanadium-loaded collagen scaffolds. Moreover, cells conserved the same proliferation rate on both kinds of membranes. Thus, toxicity was avoided when vanadium was included in the scaffold, a strategy that has been used by other groups for cytotoxic drugs [34, 39, 40]. Although in certain cases toxicity has been associated with scaffold degradation [38–40], in our present system degradation of the collagen scaffold with or without VOAsc, if it exists, does not appear to cause toxicity.

## 5. Conclusion

In conclusion, we have developed ordered collagen scaffolds for bone and cartilage tissue regeneration. VOAsc inclusion into the scaffolds effectively promoted differentiation of BMPC to osteoblasts and chondrocytes, without toxic effects. Our results indicate that the collagen-VOAsc construct could be of potential use in osteochondral tissue engineering.
